# Electromagnetic Fields and the Precautionary Principle

**DOI:** 10.1289/ehp.0901111

**Published:** 2009-11

**Authors:** Michael Kundi, Lennart Hardell, Cindy Sage, Eugene Sobel

**Affiliations:** Institute of Environmental Health, Medical University of Vienna, Vienna, Austria, E-mail: michael.kundi@meduniwien.ac.at; Department of Oncology, Örebro University Hospital, Örebro, Sweden; Sage Associates, Santa Barbara, California; Friends Research Institute, Los Angeles, California

Since Galileo, debates in science are supported by logical reasoning and reference to statements of fact and not by reference to “authorities.” Consequently, literature serves or should serve two purposes: to give credit to thoughts expressed earlier by others, and to refer to statements of facts.

The article by Dolan and Rowley (2009)— employees of the mobile telephone industry—is an example of a compilation of points of views expressed by authorities. No number of references to authoritative statements can replace scientific discourse. The article can be summarized as follows: There is no convincing evidence of harm from exposure to microwaves below levels recommended by the International Commission on Non-Ionizing Radiation Protection (ICNIRP) (1998); therefore, there is no harm, and hence application of the precautionary principle is not indicated.

Indeed, the precautionary principle is not intended as a response to unfounded fears of the public or to aim at zero risk, but as a risk management strategy in case of scientific uncertainty about the existence or magnitude of a risk. Apparently Dolan and Rowley (2009) are not aware that their subjective reasoning does not differ from the unfounded fears of the public and can be summarized as “unfounded reassurance of no harm.”

In principle, ethical considerations, value judgments, and consensus play an important role when giving guidance to public health policy. This is because “it is impossible to derive . . . a proposal for a policy from a sentence stating a fact” (Popper 1945). Use of subjective terms such as “sufficient evidence” (let alone “convincing evidence”— convincing for whom?) or “adverse effect” is unavoidable. Referring to the World Health Organization (WHO 2000), Dolan and Rowley (2009) stated: “The corresponding advice to governments is to adopt science based guidelines and not to undermine confidence by incorporating additional arbitrary safety factors.” The expression “science-based guidelines,” if taken literally, is a contradiction in terms. Although public health guidelines should be based on a thorough risk assessment, neither the assessment itself nor the reasoning that is applied to derive a guideline can be scientific. No scientific evidence can define a margin of safety; no scientific evidence can replace the value judgment of which evidence to rely on, which evidence to dismiss, and so forth. Safety factors are always—at least to certain degree—arbitrary. For example, we very rarely have scientific evidence about the distribution of sensitivity to a toxic agent in the population; therefore, we apply arbitrary factors for taking interindividual differences into account. What is important, and nearly always neglected in the area of electromagnetic fields (EMF), is to clearly state where value judgments and arbitrary decisions entered the argument and the derivation of guidelines.

The international standards for EMF (ICNIRP 1998; IEEE 2006) are based on immediate effects of exposure, such as excitation of nerve or muscle cells for low-frequency fields and increase of body temperature for high-frequency fields, not because there are no other effects, even at levels far below the guideline levels derived from these acute effects, but because the panels came to the consensus that these other effects cannot (yet) form the basis for the derivation of guidelines. For example, the International Agency for Research on Cancer (IARC 2002) classified power frequency magnetic fields as a possible human carcinogen. In that case, the subjectivity of the assessment is fully transparent: The basic rules of IARC were violated, as the panel questioned whether epidemiologic evidence can be causally interpreted in spite of evidence that neither bias nor confounding accounts for the increased childhood leukemia risk. The exposure level for which there is evidence of an increased childhood leukemia risk is far below the international standards, but the panels setting the standard did not use this evidence as a basis for the derivation of a guideline level for power frequency fields. There are surely many arguments for this decision. However, none are scientific. This is not meant as a reproach, because we recognize the fact that guidelines cannot be derived from scientific statements alone.

It would be much more appropriate if Dolan and Rowley expressly stated that they are completely satisfied with the international standards and that the industry does not want to be bothered by allusions to precaution.
